# Verifying explainability of a deep learning tissue classifier trained on RNA-seq data

**DOI:** 10.1038/s41598-021-81773-9

**Published:** 2021-01-29

**Authors:** Melvyn Yap, Rebecca L. Johnston, Helena Foley, Samual MacDonald, Olga Kondrashova, Khoa A. Tran, Katia Nones, Lambros T. Koufariotis, Cameron Bean, John V. Pearson, Maciej Trzaskowski, Nicola Waddell

**Affiliations:** 1 Max Kelsen, Brisbane, QLD 4006 Australia; 2 QIMR Berghofer Medical Research Institute, Brisbane, QLD 4006 Australia; 3 Queensland University of Technology, Brisbane, QLD 4000 Australia

**Keywords:** Computational biology and bioinformatics, Computational models, Data processing, Machine learning

## Abstract

For complex machine learning (ML) algorithms to gain widespread acceptance in decision making, we must be able to identify the features driving the predictions. Explainability models allow transparency of ML algorithms, however their reliability within high-dimensional data is unclear. To test the reliability of the explainability model SHapley Additive exPlanations (SHAP), we developed a convolutional neural network to predict tissue classification from Genotype-Tissue Expression (GTEx) RNA-seq data representing 16,651 samples from 47 tissues. Our classifier achieved an average F1 score of 96.1% on held-out GTEx samples. Using SHAP values, we identified the 2423 most discriminatory genes, of which 98.6% were also identified by differential expression analysis across all tissues. The SHAP genes reflected expected biological processes involved in tissue differentiation and function. Moreover, SHAP genes clustered tissue types with superior performance when compared to all genes, genes detected by differential expression analysis, or random genes. We demonstrate the utility and reliability of SHAP to explain a deep learning model and highlight the strengths of applying ML to transcriptome data.

## Introduction

The application of artificial intelligence (AI) via machine learning (ML) algorithms to medical data has the potential to improve healthcare systems and the ability to complement or outperform clinical experts^1,2^. To date, the use of AI in healthcare has largely focused on visual data, such as radiology^3–5^ and histology imaging^1,6^, as ML outputs can be visually confirmed by medical professionals. A strength of AI is the ability to integrate complex data for patient diagnosis; recently, an approach to integrate molecular and genomic features was developed to identify early-stage lung cancer patients from risk-matched controls^7^. However, deep learning algorithms such as convolutional neural networks (CNNs) have convoluted statistical structures with multiple hidden layers and non-linear activation functions, making the predictions of these models difficult to interpret and explain. The lack of transparency or explainability of ML algorithms means conforming to regulatory standards is challenging, and prevents their widespread adoption in healthcare.

Explainability models are methods capable of explaining ML algorithms that can provide robust replicable reasoning for the features which are important to the ML’s decision-making process. A number of methods have been proposed to make a range of ML models, particularly deep neural networks, explainable, including Shapley Sampling^8^, Relevance Propagation^9^, Quantitative Input Influence^10^, LIME^11^, and DeepLIFT^12,13^. In 2017, Lundberg and Lee^14^ unified these methods and used the principles of fair distribution of payoffs from game theory proposed by Lloyd Shapley^15^ to ensure a link between local explainability (the feature contribution of a single feature within a single sample) and global explainability (feature contribution of the whole model). This proposed framework for explaining predictions of ML models is now known as SHAP (SHapley Additive exPlanations)^14,16^. SHAP assigns an importance value—known as a SHAP value—to each feature, where positive values indicate an increase in the probability of a class, and negative values reduce the probability of the class.

In this study, we set out to demonstrate the reliability of the SHAP explainability model, specifically the SHAP GradientExplainer, for high-dimensional data. First we trained a CNN model to classify tissue types on public RNA-seq data from the Genotype-Tissue Expression (GTEx) project^17^—the largest comprehensive public transcriptome dataset of non-diseased tissue-specific expression to date. Once the performance of the CNN was determined, we used SHAP values to identify the most discriminatory genes and compared them to genes identified as differentially expressed (edgeR^18^). Finally, we validated the stability of SHAP values by replicating the outcome in an independent dataset.

## Results

### Training a CNN to predict tissue type using RNA-seq data as a basis for SHAP

**Figure 1:**
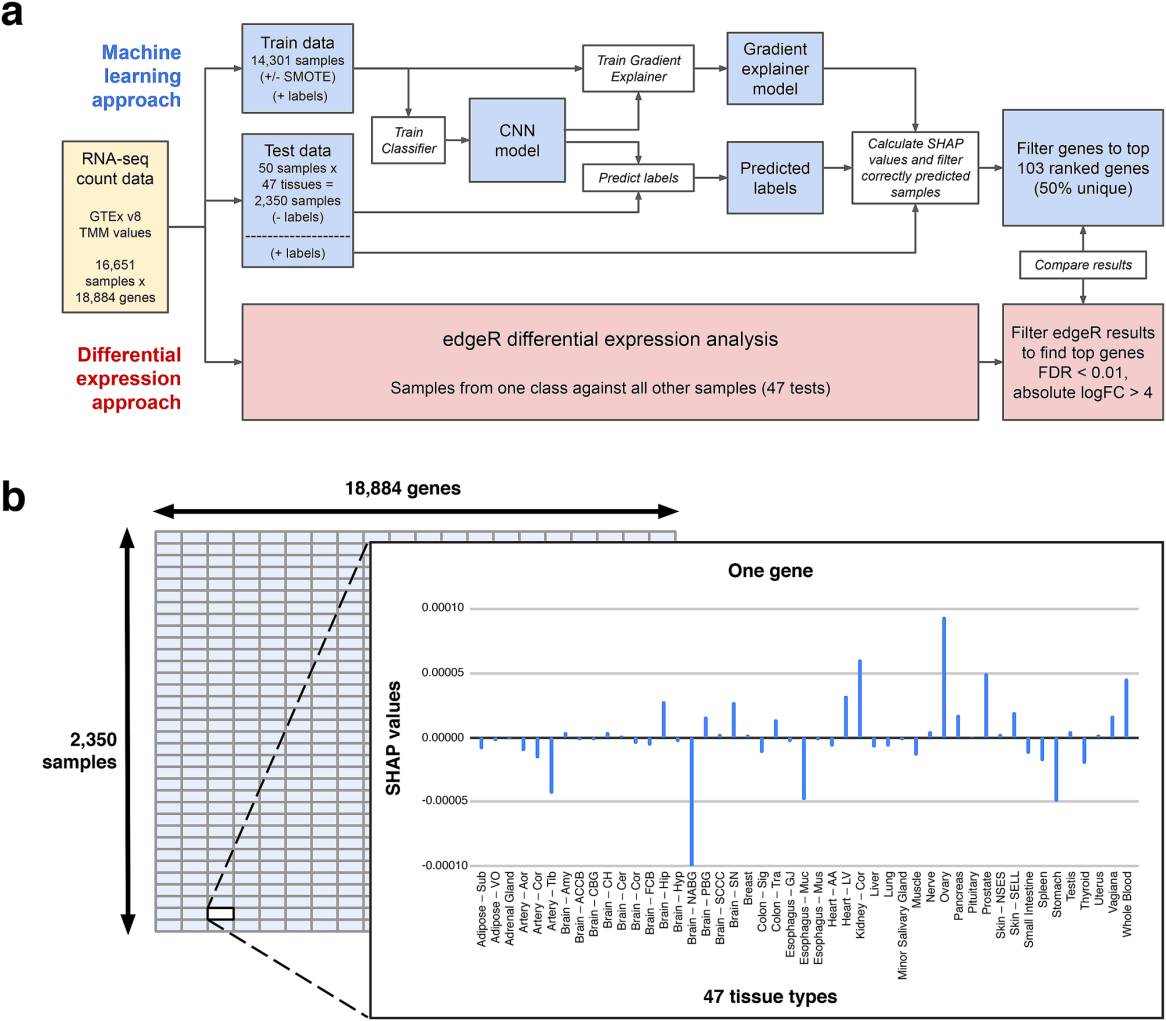
Study overview. (**a**) Genotype-Tissue Expression (GTEx) RNA-seq data were analysed in parallel using an ML approach to generate SHapley Additive exPlanation (SHAP) values (blue), and using a differential expression analysis approach (red). (**b**) Schematic showing the shape of SHAP output for held-out test data. Matrix of 18,884 genes × 2350 samples, where each cell contains values for each tissue type making the matrix 18,884 × 2350 × 47. The inner schematic shows SHAP values that represent the contribution of the gene to the prediction, where positive SHAP values indicate a positive effect on the model’s confidence in the tissue type label, and negative SHAP values indicate a negative effect. When filtering SHAP values, only the SHAP value corresponding to the correct tissue type was selected and the remaining values were omitted, making the final matrix 18,884 × 2,350. CNN: Convolutional Neural Network; FDR: False Discovery Rate; logFC: log2 Fold Change ; SMOTE: Synthetic Minority Over-sampling Technique; TMM: Trimmed Mean of M-values.

We utilised RNA-seq data from the GTEx project in the form of Trimmed Mean of M-values (TMM) for 18,884 genes (features) derived from 16,651 samples and 47 tissue types (classes; Supplementary Table S1). An ML approach and a differential expression approach were used to analyse the data, outlined in Fig. 1a. For the ML approach, 50 samples from each tissue type were randomly selected as a held-out test set (n = 2350), and the remaining samples comprised the training set (n = 14,301) for a CNN to predict tissue type. We used the explainability method SHAP to identify the important features (genes) driving the CNN learning process, and compared the genes to those from the differential expression analysis approach (Fig. 1a). The input to the CNN was an 18,884-gene vector that was converted into a matrix, where for each sample a network of various hidden layers outputs a prediction of one of the 47 tissue types (Supplementary Fig. S1a, b). SHAP values were calculated for each gene and each sample in the held-out test set (Fig. 1b).

### Performance of the CNN is similar using imbalanced or balanced training data

**Figure 2:**
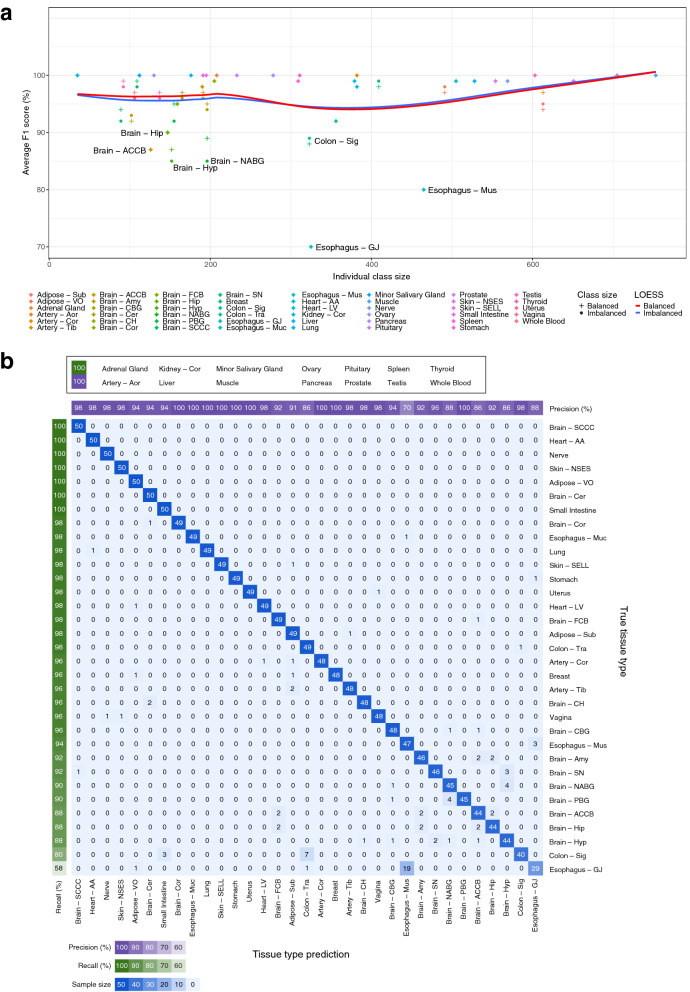
Convolutional neural network (CNN) performance using imbalanced and balanced class sizes. (**a**) Comparing CNN performance (macro average F1 scores, y-axis) using class-imbalanced (filled circle) and Synthetic Minority Over-sampling Technique (SMOTE) class-balanced (plus symbol) training sets. Sample size (x-axis) represents class-imbalanced tissue sample size; all class-balanced tissues consist of 752 or 753 samples, so are displayed here as equal to the respective class-imbalanced class size to compare F1 scores within the same class. The blue and red lines represent regression line fits using LOESS regression for the imbalanced and balanced datasets, respectively. Tissue types with F1 scores ≤ 90% are labelled. (**b**) Confusion matrix displaying recall and precision of the CNN model trained on SMOTE class-balanced data. The CNN model was evaluated using held-outGenotype-Tissue Expression (GTEx) data (50 samples per tissue type). Each row corresponds to the true tissue type, and each column corresponds to the CNN tissue type prediction. Recall and precision values are shown along the left (green) and top (purple) sides of the matrix, respectively, where tissue types are ordered by recall. Tissue types with 100% recall and precision are listed in the top panel, and are not shown in the confusion matrix.

The number of samples per tissue type in the training set ranged from 35 for Kidney—Cortex to 753 for Muscle—Skeletal (Supplementary Fig. S2a, Supplementary Table S1). We assessed the impact of balanced and imbalanced training sample sizes on CNN performance for the held-out test data (Fig. 2a, Supplementary Fig. S2b). CNN performance was measured using the F1 score, which is the harmonic mean of precision and recall. The Synthetic Minority Over-sampling Technique (SMOTE) of over-sampling the data to match the largest class size produced the highest F1 scores for predicting tissue type and outperformed imbalanced data and other sampling approaches including Random Under-Sampling (RUS) and Random Over-Sampling (ROS; Supplementary Fig. S2b). The macro-average F1 score of the CNN to predict tissue type using imbalanced data was 95.31%, and for data balanced with SMOTE was 96.10%. Similar F1 scores were obtained when comparing SMOTE-balanced training data with imbalanced training data per class, however classes with smaller sample numbers (< 200) achieved slightly higher F1 scores when class sizes were balanced across the cohort (Fig. 2a). Due to the performance improvements, we chose to continue with the CNN trained using SMOTE-balanced data. Per tissue, this classifier had recall ranging from 58 to 100% and precision ranging from 70 to 100% (Fig. 2b). Tissues with the lowest recalls were Esophagus—GJ (58%) and Colon—Sig (80%), with 38% (n = 19) of the Esophagus—GJ samples being predicted as Esophagus—Mus, and 20% (n = 10) of the Colon—Sig samples being predicted across Colon—Tra and Small Intestine. Other tissues with < 90% recall after balancing included Brain—Hyp, Brain—Hip and Brain—ACCB, where all three tissue types achieved 88% recall, and their respective misclassifications were other brain tissue types.

To validate the CNN, we assessed its performance using an independent dataset of 126 samples representing 21 tissues (3–9 samples per tissue) from the Human Protein Atlas (HPA). All tissues were classified correctly except for five adipose tissue samples that were classified as breast (Supplementary Data 1). Of note, the HPA adipose tissue samples clustered together with GTEx breast samples using Uniform Manifold Approximation and Projection (UMAP) (Supplementary Fig. S3). This finding may be explained by the source of the HPA adipose tissues, as two of the five samples were collected from breast and the rest were from abdominal fat.

### Identification of the most explainable genes using SHAP

**Figure 3:**
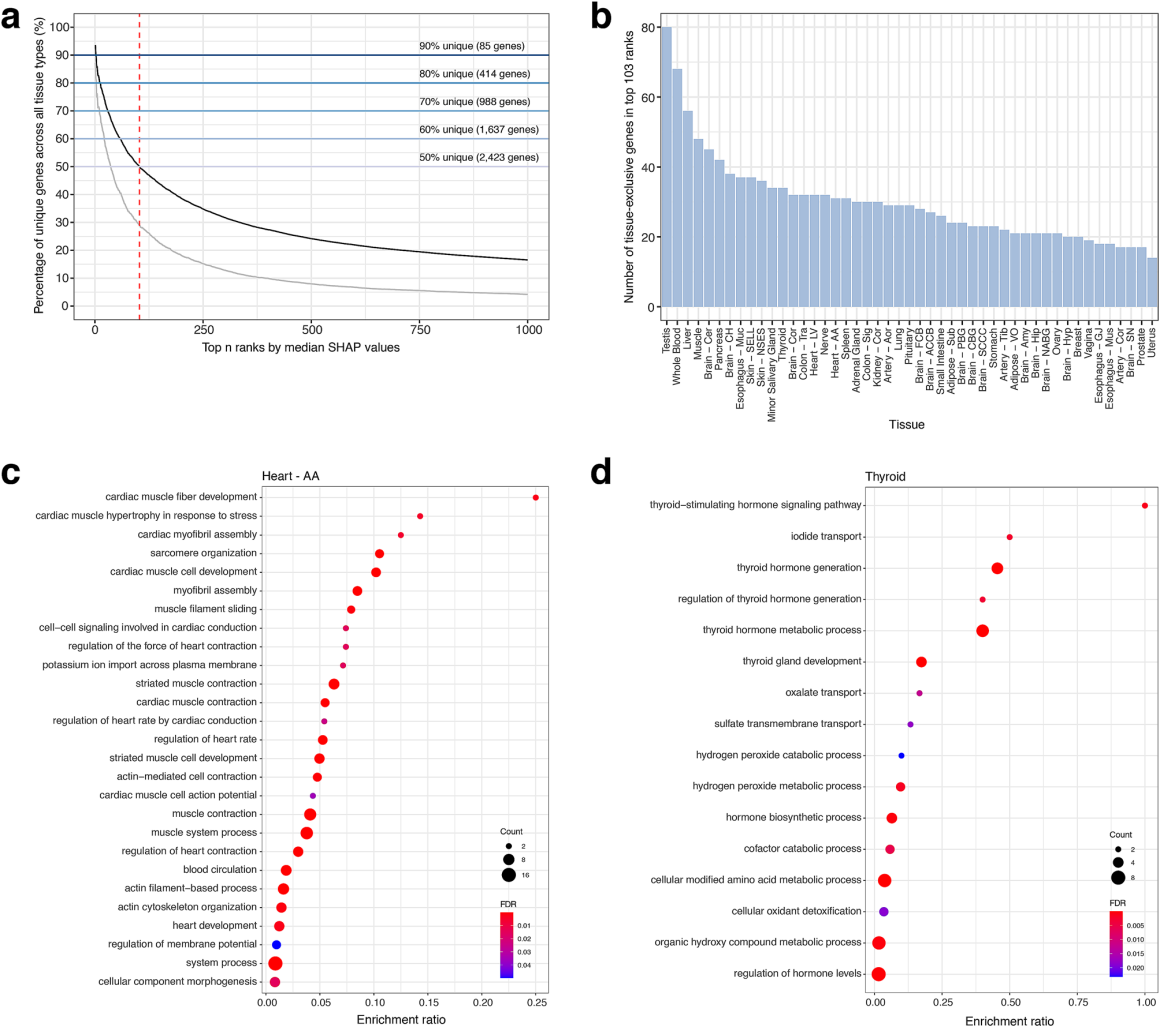
Identifying the most salient genes from the Convolutional neural network (CNN) model using SHapley Additive exPlanations (SHAP) for each tissue type. Median SHAP values were calculated per gene per tissue, then gene ranks were determined based on SHAP values per tissue. (**a**) Line plot showing the decreasing percentage of unique genes (black line) and tissue-exclusive genes (grey line) in expanding top n ranks of possible genes across all 47 classes (y-axis). The top 103 ranks (red dashed line) contain a total of 4,841 genes (47 classes × 103 ranks = 4,841), but some genes occur in multiple tissues, with approximately 50% of genes (2,423 genes) being unique. (**b**) The number of tissue-exclusive genes in the top 103 ranked genes per tissue type. Enriched Gene Ontology (GO) biological processes using tissue-exclusive genes for (**c**) Heart—AA and (**d**) Thyroid. Enrichment ratio was computed as the ratio of the observed gene count to background gene count.

To identify the features driving the CNN learning process, we calculated the median SHAP values associated with each gene across all correctly predicted test samples within each tissue. We identified the most salient genes for differentiating the 47 tissue types by ranking each gene by median SHAP value within each tissue. Of the 47 genes in the first rank, 93.6% (44 out of 47) were unique (if a gene was found in > 1 tissue it was only counted once), and 87.2% (41 out of 47) were tissue exclusive (present in only one tissue). The top 103 ranks contained a total of 4,841 genes (47 classes × 103 ranks = 4,841), with approximately 50% of genes (2,423 genes; termed ‘SHAP genes’) being unique, and 29.1% (1407 genes) of genes being tissue exclusive (Fig. 3a, Supplementary Data 2). The number of tissue-exclusive genes varied by class, with Testis containing the highest number of exclusive genes (80 genes) and Uterus containing the lowest (14 genes; Fig. 3b).

To test the stability of SHAP values, we identified the top 103 ranked genes for the 21 tissues from the independent HPA dataset (Supplementary Data 3). Encouragingly, the overlap of the SHAP genes between GTEx and HPA test samples ranged from 20 to 63 genes (median 41 genes) for the matched tissues (Supplementary Fig. S4).

To assess the biological relevance of the SHAP genes, we first examined the number of protein–protein interactions per tissue using STRING v11^19^. All tissues had a significantly higher number of interactions among the proteins for the SHAP genes than at random, and 34 of 47 tissues had a significantly higher number of interactions for the tissue-exclusive SHAP genes (Supplementary Data 4). We then assessed the biological processes enriched in these gene lists. Gene Ontology (GO) biological processes that were commonly enriched in the SHAP genes included organ morphogenesis, regionalization, and organ development (Supplementary Data 5). Furthermore, processes enriched in tissue-exclusive genes largely reflected biological processes important for those tissues (Supplementary Data 6). For example, enriched processes for heart—atrial appendage (Heart—AA) included cardiac muscle fiber development and cell–cell signaling involved in cardiac conduction, while pathways for thyroid gland (Thyroid) included iodine transport and thyroid hormone generation (Fig. 3c,d).

### The genes identified by SHAP are similar to the significant genes identified by differential expression analysis

To compare the SHAP genes (2,423 genes) to a non-ML approach, we performed differential expression analysis on the GTEx RNA-seq data, as outlined in Fig. 1a. The non-ML approach used edgeR to compare gene expression of samples in one class to the remaining samples, then filtered for significant genes according to absolute log2 fold change (logFC) > 4 and false discovery rate (FDR) < 0.01. The total number of significantly differentially expressed genes identified by edgeR was 30,532, where some genes were identified more than once across tissues. Testis showed the greatest number of up-regulated genes, and Whole Blood showed the greatest number of down-regulated genes, consistent with previous reports^20^ (Supplementary Fig. S5a). The number of significantly differentially expressed tissue-exclusive genes varied greatly by class, with the majority exclusive to Whole Blood and Testis, and five tissues (Artery—Cor, Brain—ACCB, Brain—Hip, Esophagus—GJ, and Vagina) without any tissue-exclusive genes (Supplementary Fig. S5b).

**Figure 4:**
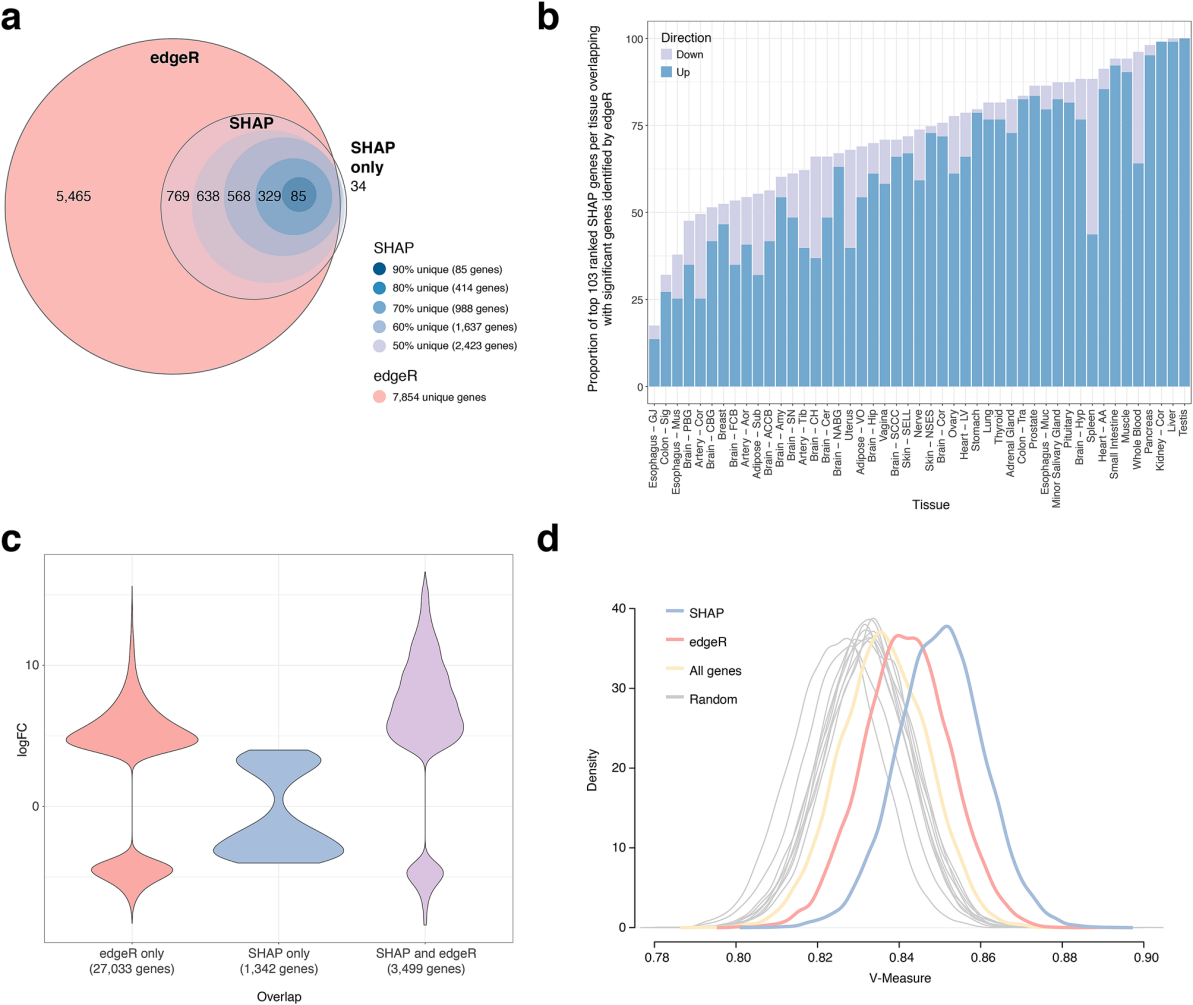
Comparison of the most salient genes from the Convolutional neural network (CNN) model according to SHapley Additive exPlanations (SHAP) and the significant genes from edgeR. (**a**) Euler diagram comparing the unique sets of gene lists from edgeR (7,854 genes) and SHAP, where the outermost circle for SHAP represents the unique genes from top 103 ranks (2,423 genes; 50% unique), and the circles within, from largest to smallest, represent the unique genes from the top 58 (1,637 genes; 60% unique), 30 (988 genes; 70% unique), 11 (414 genes; 80% unique) and 2 (85 genes; 90% unique) ranks, respectively. (**b**) The proportion of the top 103 ranked genes according to SHAP that overlap with all significant edgeR genes per tissue. Direction of expression of each gene per tissue was obtained from edgeR analyses, where up (blue) represents genes with log2 Fold Change (logFC) > 4, and down (lilac) represents genes with logFC < -4. (**c**) Violin plots of the top genes unique and common to the edgeR and SHAP methods at the individual tissue level, showing logFC as determined by edgeR. Some genes are included more than once if they were identified in multiple tissues. The SHAP method identified 4841 top genes (top 103 ranked genes, 50% unique), and the edgeR method identified 30,532 significant genes. The SHAP and edgeR methods had 3499 genes in common ("SHAP and edgeR", purple). Thus, 30,532–3499 = 27,033 genes were unique to the edgeR method ("edgeR only", red) and 4841–3,499 = 1342 genes were unique to the SHAP method ("SHAP only", blue). (**d**) V-Measure of k-means clustering analysis on Uniform Manifold Approximation and Projection (UMAP). k-means clustering was performed using SHAP genes (2423 genes), 10 random sets of 2423 genes, edgeR genes (7854 genes), and all genes (18,884 genes).

To compare edgeR results to the genes identified by SHAP, we collapsed the significantly differentially expressed edgeR genes across all tissues to determine the unique genes; a total of 7,854 genes were found to be unique using edgeR (termed ‘edgeR genes’). A comparison between the edgeR and SHAP genes collapsed across all tissues revealed that 98.6% of the SHAP genes overlapped with the genes identified by edgeR, with only 34 SHAP genes not identified as significant by edgeR (Fig. 4a). Pathway analyses of the 2423 SHAP genes and 7854 edgeR genes revealed similar pathways, including enrichment in GPCR ligand binding, neuronal system, extracellular matrix organization and keratinization pathways (Supplementary Fig. S6a, b). At the individual tissue level, when we compared the top 103 ranked genes identified by SHAP per tissue (4841 genes) with the most significant genes identified by edgeR within each tissue type (30,532 genes), we found the overlap amount varied, with 100% overlap in Testis, and < 25% overlap in Esophagus—GJ (Fig. 4b). In total, 1342 of the top 103 ranked genes identified by SHAP per tissue did not pass edgeR filters, as the majority of these genes (98.3%) passed the FDR filter but not the logFC filter (Fig. 4c). Interestingly, the genes that overlapped between edgeR and SHAP were more frequently up-regulated than down-regulated (Fig. 4b,c).

To determine the classification properties of the genes identified by SHAP, we investigated how well the 2423 SHAP genes discriminate tissue type by clustering all test samples (n = 2350) using UMAP, and compared the results to UMAPs based on all 18,884 genes, the genes detected by edgeR, and random subsets of 2423 genes (Supplementary Fig. S7). We evaluated the quality of UMAP embeddings with k-means classification using the V-Measure, which is the harmonic mean of homogeneity and completeness; homogeneity tests whether a cluster contains only members of a single class, and completeness tests whether all members of a given class are assigned to the same cluster. The SHAP genes obtained the highest V-Measure (0.850), outperforming edgeR (0.842), all genes (0.836), and 10 random subsets of 2423 genes (0.824–0.834; Fig. 4d), although the difference was not significant. To test whether the clustering achieved with the SHAP genes (measured using mean V-Measure) could have been achieved if genes were picked at random, we built a “null” distribution of mean V-Measures from 100 random subsets of 2,423 genes (Supplementary Fig. S8). The probability of achieving the high mean V-Measure by chance compared to the one achieved with the SHAP genes (0.850) was very low (*P* < 9.0e−07 by one-tailed Student’s t-test).

## Discussion

A major criticism of AI is that it is a “black box” as we cannot identify or explain the features driving its decisions. Earlier attempts to explain ML models can be vulnerable towards the model and data generating process^9^, input perturbations^21^, and instability between local and global features^14^. SHAP is a unified approach for explaining ML model predictions that addresses the limitations of other methods, including the lack of stability between the explainability of the local and global features. SHAP was recently used within the MethylNet deep learning tool to discover important methylation states in different cell types and cancer subtypes^22^, and within the DEcode deep learning tool to determine key regulators of differential expression across human tissues^23^. Nonetheless, the reliability of SHAP in complex, high-dimensional data remains unknown. In this study, we examined the reliability of the SHAP explainability method applied to a complex black box model. We trained a CNN model to classify tissues using the largest publicly available transcriptome dataset, GTEx v8. We then used SHAP to explain and identify key features driving the classifier, and show that across all tissues, the large majority of the most relevant features were also identified by differential expression analyses. We show that the clustering achieved with the SHAP genes has a low probability of being achieved when genes are randomly selected, further increasing our confidence in the selection of meaningful features that explain the CNN’s decision-making during classification.

A valuable application for explainable deep learning models is healthcare, whereby imaging, genome and transcriptome data may be used to infer clinical outcomes such as prognosis and tumour type classification. The GTEx project is a large multi-class resource that allows us to establish a deep learning model for tissue classification and test explainability models. Classification of a variety of tissue types has been successfully achieved using traditional ML models^24,25^, and led to the identification of major tissue-specific gene signatures^20,25^. Previously, GTEx-trained classifiers were created using random forests and linear models^24,25^. However, we cannot directly compare our CNN classifier to these existing classifiers, as the previous studies used earlier GTEx data releases (V4 and V6) compared to our study (V8), and only predicted the respective number of major GTEx tissue types and not the 47 tissue subtypes predicted herein. Our main aim was not to outperform previous tissue classifiers; however, we show that our CNN model obtained high performance scores on held-out GTEx samples and an independent dataset, thus explaining the decision driving features of such a model was worthwhile. The most poorly predicted tissues were similar tissue subtypes, which are likely to contain similar cell types. For example, a subset of Colon—Sig samples were predicted as Colon—Tra; both tissue types are part of the same organ (colon), they have similar cell type composition, and it has been shown that gradual changes in transcript expression occur along the colon from the cecum to the rectum^26^. Similarly, the Esophagus—GJ and Esophagus—Mus were frequently predicted as each other, which can be explained by the fact that the esophagus-gastroesophageal junction contains a large amount of muscle tissue and cell types that comprise the esophagus muscularis layer. Finally, misclassifications were observed between brain tissues. This is not surprising as most brain samples have been shown to cluster together^27^, and the differences between brain subclasses may have been more apparent if differential transcript splicing was taken into consideration^20^. Interestingly, the two brain tissue types that were sampled twice, once at autopsy and later at the biobank (cerebellum, Brain—Cer and Brain—CH; frontal cortex, Brain—Cor and Brain—FCB), had minimal misclassification between the respective collections, which, in agreement with previous findings, indicates that sample collection time can affect gene expression^28^.

No standard approach for selecting the most relevant features identified by the explainability models has yet been defined. To identify the key features driving our CNN tissue classifier, we chose to select the top 103 ranked genes according to median SHAP value per tissue as, at this rank, 50% of the genes across all tissues were identified as unique. However, a standard approach for determining the most relevant features, particularly in the context of feature-additive models such as SHAP, is yet to be optimised. Improvements to further optimise SHAP calculations and deep learning models should also be considered as they are still computationally expensive. For example, we found DeepExplainer not tractable in the context of high-dimensional data. Although simpler methods exist, such as TreeExplainer^29^, their utility in biological data might be limited by the simplicity of the model driving learning and prediction. Furthermore, we expect that incorporating the full transcriptome, not limited to protein-coding transcripts, may improve the classification and feature detection.

To determine whether the SHAP explainability model identified plausible features, we compared the SHAP genes to the results from differential expression analysis, which was used as a reference to assess the correctness of estimated feature relevance according to assigned SHAP values. The edgeR method is designed to identify significantly differentially expressed genes between two groups; it is not designed to be a classifier and nor do we use it this way. As in many other gene expression studies, we applied a fold change cut-off under the assumption that small expression differences may not be biologically meaningful. Interestingly, the SHAP method identified a small subset of genes that were unique to SHAP, with more subtle impact on gene expression changes, which could indicate that these genes are biologically meaningful in the context of tissue classification. Thus, unlike differential expression analysis, deep learning classifiers may detect more subtle gene signatures and intricate gene co-expression relationships and thus has the potential to find novel insights.

Tissue-exclusive gene expression occurs due to the different composition of cell types within each tissue. Individual cell types can be marked by specific methylation patterns^30^ that drive gene expression. We found that a relatively small proportion of the top tissue discriminating genes identified through SHAP values were tissue exclusive, and that these numbers quickly declined leading to a strong domination of genes common across classes. As previously discussed, some tissues may contain similar cell types and additionally may have shared biological processes. Another potential explanation is the notion of genetic pleiotropy, where one gene can affect multiple traits, and thus a gene can have multiple roles in different cell types. Some tissues contained a large proportion of tissue-exclusive genes as detected by both SHAP and edgeR methods, with testis showing the highest number of exclusive genes, which agrees with a previous study that found the largest number of tissue-enriched genes was in testis, followed by brain and liver^31^. The differences in expression between some tissues were so marked that even when we used all genes and random genes to cluster samples, relatively good separation between the tissue types was observed. Therefore, it is not surprising that the CNN could predict tissue type with high recall and precision and performed well on an independent dataset.

A challenge for ML models is small and/or imbalanced training datasets. Under-represented classes might not have sufficient data, resulting in models with biased representations. Similarly, over-represented classes might dominate the explainability by promoting genes that, although good tissue type discriminators, are not equally important to less represented tissues. We tested several approaches to mitigate the class imbalances in the GTEx dataset, including re-sampling and over-sampling. We showed that SMOTE^32^, which was previously shown to improve classifier predictions in high-dimensional class-imbalanced data^33^, slightly improved the predictive performance of the CNN compared to other class balancing approaches. The improvement was particularly beneficial to the smaller classes in terms of their prediction accuracy.

In summary, we demonstrate the reliability of SHAP in the context of deep learning classifiers trained on RNA-seq data. We show that SHAP values used to explain a CNN can identify genes comparable to a non-ML approach and reveal biological meaningful insights and can generalise to independent data. Our work shows that explainability models will facilitate the use of deep learning in biological and clinical data and have the potential to provide novel insights. The continued development of high-performance classifiers together with reliable methods that explain feature importance will allow domain experts to rule out spurious features and pave the way towards safe use of AI in healthcare.

## Methods

### Ethics approval

This work was approved by the QIMR Berghofer Human Research Ethics Committee (P2095).

### RNA-seq data

GTEx analysis V8 RNA-seq raw gene read counts and gene Transcripts Per Million (TPM) were downloaded from the GTEx Portal (https://gtexportal.org/home/datasets). The RNA-seq data consisted of 17,382 samples from 948 donors across 54 tissue types, and expression values for 56,200 genes. Tissue types with fewer than 25 samples (Bladder, Cervix—Ectocervix, Cervix—Endocervix, Fallopian Tube, and Kidney—Medulla) and data derived from cell lines (Cells—Cultured fibroblasts and Cells—EBV-transformed lymphocytes) were excluded from our analyses. The remaining 16,651 samples from 47 tissue types were used in further analysis (see Supplementary Table S1 for tissue type abbreviations).

RNA-seq FASTQ data for the independent HPA dataset were downloaded from the ENA (Study ID PRJEB6971). Reads were trimmed for adapter sequences using Cutadapt version 1.9^34^ and aligned using STAR version 2.5.2a^35^ to the human reference genome GRCh38/hg38. Genes were annotated using GENCODE v26 collapsed as per GTEx methods. Expression was estimated using RSEM version 1.2.30^36^. HPA tissue type labels were manually annotated with GTEx tissue type labels (Supplementary Data 1).

### Filtering and normalization of RNA-seq data

All RNA-seq data filtering and normalization steps were performed in R version 3.5.1. The RNA-seq data were filtered to include protein-coding genes and genes that were considered expressed. Specifically, for raw gene counts, genes were kept if they contained >  = 6 reads in > 80 samples, and for gene TPM counts, genes were kept with > 0.1 TPM in > 80 samples. The number of samples was informed by the tissue with the smallest sample size which was Kidney—Cortex (n = 85), therefore, genes that appeared in at least 80 samples were kept. To allow a comparison between raw gene counts and TPM values, the intersection of the filtered gene count and TPM lists was used in all analyses and resulted in 18,884 protein-coding genes. TMM normalization was performed on the filtered raw gene counts using the edgeR package 3.24.0^18^. Although we had access to both TPM and TMM normalized values, to maintain direct comparison with edgeR approach we continued with TMM values for further analyses. The raw gene counts from the HPA data were filtered to include the same 18,884 protein-coding genes used to train the CNN model, and TMM normalization was performed as above.

### Splitting train and test data

All ML methods were performed using Python version 3.6, sklearn version 0.21.1, and Keras version 2.2.4. To split the data into train and test datasets, 50 samples from each tissue type were randomly selected to form a “held-out” test dataset (n = 2350 total) with the remaining 14,301 samples used as the training dataset. Only one test dataset was used for all validation analyses. The number of held out samples for that dataset was arbitrary and selected to maximize the size of the training sample while at the same time producing a held-out dataset which would have enough samples per tissue for accurate estimates of prediction.

### Over-sampling with SMOTE

To generate a balanced training dataset with equal numbers of samples per tissue type, we over-sampled under-represented classes to match the number of the largest class using SMOTE^32,33^. Among the 47 tissue types, the number of training samples per tissue type ranged from 35 for Kidney—Cortex to 753 for Muscle—Skeletal. SMOTE created new synthetic data using the *k*-nearest neighbours method. For each original sample, the four or five nearest neighbours were selected, and one new sample was synthesised in the direction of each of those neighbours. This created a balanced training dataset of 35,362 samples. SMOTE was performed using the MulticlassOversampling function from the smote-variants library version 0.3.4^37^.

### Transforming gene expression values for input to CNN model

We applied the following transformation for TMM values (where x is the gene expression input vector) to reduce the bias of outliers and reduce the quantity of zeroes that the CNN’s ReLU activations would be unable to propagate across:

1 $${\log_2}\left( {x + 0.001} \right) + 10$$

### CNN

We trained a CNN using imbalanced and balanced datasets of 18,884 genes from 14,301 (imbalanced) and 35,362 (balanced) samples. A CNN requires input data in a two-dimensional matrix format, therefore a zero-padding to gene vectors was applied (18,884 increased to 19,044) to reshape them into 138 by 138 square matrices. The CNN included 4 back-to-back convolutional blocks, each consisting of a convolutional layer with a 3 × 3 convolutional kernel, followed by batch normalization and ReLU activation. Max pooling was applied after the second and fourth convolutional blocks to reduce the size of the features vector by half. After the last convolutional block, a 34 × 34 × 256 features vector was produced for each input and then flattened into a 295,936 × 1 embeddings vector. Two fully connected layers were then applied to this embeddings vector to gradually reduce the size to 47 × 1, finally arriving at classification softmax layer (Supplementary Fig. S1b). All ML analyses were performed on Nvidia Tesla P100 GPU with a memory capacity of 16 Gigabytes.

### Evaluating the predictive performance of CNN

The performance of both the balanced and imbalanced CNN models was evaluated using the held-out test dataset. Since prediction accuracy was not an adequate index to evaluate the impact of balancing classes, we chose F1 score as an evaluation statistic, which calculates the harmonic mean between precision and recall, making it more robust against Type 1 (false positives) and Type 2 error (false negatives):

2 $$F1 = \frac{2(precision \times recall)}{precision + recall}$$

### Determining SHAP values

To identify features that were driving the CNN learning processes, we used the GradientExplainer function from the SHAP library (version 0.32.1)^16^ with the training set as the background data. The architecture and trained weights of the CNN model were saved into JSON and HDF5 files, respectively, using the Keras library, and in conjunction with the training dataset were used to initialise the SHAP explainer. The test dataset was then input into the initialised SHAP explainer and an array of 47 × 138 × 138 SHAP values was calculated for each sample. The first dimension of the output corresponds to SHAP values for each possible prediction. Inference was run on the test set to obtain the model’s predicted labels for each sample. When filtering SHAP values, only correctly predicted samples were kept in the SHAP array, and only the corresponding SHAP value of the correct class was selected, leaving a single value per gene per sample. To determine the global saliency, median SHAP value was calculated for each gene per tissue, then ranked from largest to smallest value. The process of determining SHAP values for the independent HPA dataset was performed as above, with the exception of HPA adipose tissue where SHAP values for predictions of breast class were used.

### Differential gene expression analysis

We performed tissue differential gene expression analysis in R by comparing the samples from a given tissue to the average of all of the samples that did not belong to the tissue. Differential expression analysis was performed on normalized TMM values using edgeR^18^. We used false discovery rate (FDR) < 0.01 and absolute log2 fold change (logFC) > 4 as a cut-off for statistical significance.

### Cluster analysis

To evaluate the classification properties of the SHAP genes on the held-out GTEx samples (n = 2350), we applied k-means clustering to the unlabeled UMAPs generated using umap-learn version 0.3.8 and calculated their respective V-measure scores. The number of clusters (k) used was 47, which is equal to the number of classes. This was crucial for correct benchmarking of the measures of completeness and homogeneity. The V-measure is the harmonic mean of completeness and homogeneity, where completeness measures to what extent all samples of the same class reside in a single cluster, and homogeneity measures whether each cluster contains samples from a single class^38^. The same workflow was performed using SHAP genes (2423 genes), edgeR genes (7854 genes), all genes (18,884 genes), and randomly selected gene subsets matching SHAP gene size (2423 genes).

We ran two distinct methods of cluster analysis. In the first method, where we aimed to measure the uncertainty of k-means, we ran 10,000 iterations on each of the gene subsets to obtain a distribution of k-means scores, where 10 randomly selected gene subsets matching the SHAP gene size were used. V-measures were then calculated for the k-means clustering produced by each iteration. For the second method, to estimate the probability of randomly selecting a gene subset that performs as well as the SHAP genes, we ran 100 random gene samplings, each with 100 random k-means initialisations. The mean of each k-means sampling distribution was then selected. Consequently, we ended up with 100 values from each of the random gene selections. We used these 100 values (100 means) to build the null distribution. The mean values from the SHAP k-means sampling therefore became our “true” test statistic. We performed a one-tailed Student t-test to estimate the probability of selecting the SHAP genes by chance.

### Protein–protein interactions and pathway analysis

Protein–protein interaction analysis was performed using STRING v11 using the online portal. All interaction sources were included and medium confidence (0.4) interaction threshold was used. Gene names were converted to protein names using STRING-db text matching. Only query proteins were considered. GO Biological processes, KEGG^39^ and Reactome pathway analysis results were downloaded from STRING-db online portal.

Pathway analysis was performed using ReactomePA R/Bioconductor package version 1.26.0^40^. The bitr function from the clusterProfiler package version 3.10.0^41^ was used to convert gene IDs from Ensembl to Entrez, then consequently passed to the ReactomePA enrichPathway function, before plotting the results with the clusterProfiler dotplot function.

### Data visualisation

Figure 1a,b and Supplementary Fig. S1a were generated using Google Slides. Figures 2a, 3a–d, 4b,c, Supplementary Fig. S2b, Supplementary Fig. S3a,b, Supplementary Fig. S4, and Supplementary Fig. S5a,b were generated using ggplot2 version 3.2.1^42^. Figure 2b and Supplementary Fig. S2a were generated using Google Sheets. Figure 4a was generated using eulerr version 5.1.0. Figure 4d, Supplementary Fig. S7a-d, and Supplementary Fig. S8 were generated using matplotlib version 3.1.0. Supplementary Fig. S1b was generated using Lucidchart. Supplementary Fig. S6a-b were generated using clusterProfiler version 3.10.0. Final figure formatting was performed using Adobe Illustrator.

## Supplementary Information

Supplementary Information 1.

Supplementary Information 2.

Supplementary Information 3.

Supplementary Information 4.

Supplementary Information 5.

Supplementary Information 6.

Supplementary Information 7.

## Data Availability

No new data were generated in this study. RNA-seq gene read counts from GTEx analysis release v8 were downloaded from GTEx Portal (https://gtexportal.org/home/datasets).

## Code availability

Tools and code that were used in this publication are available from GitHub at https://github.com/MaxKelsen/GradientExplainer-RNAseq.
