# Supplementary material for: Verifying explainability of a deep learning tissue classifier trained on RNA-seq data
Source: Sci Rep. 2021 Jan 29;11:2641. doi: 10.1038/s41598-021-81773-9 (PMC7846764; doi:10.1038/s41598-021-81773-9)
Supplement: Supplementary file 7 — Supplementary Information 7. [file 41598_2021_81773_MOESM7_ESM.docx]

**Supplementary Information**

# **Verifying explainability of a deep learning tissue classifier trained on RNA-seq data**

# Melvyn Yap^1^^, Rebecca L. Johnston^2^^, Helena Foley^1^^, Samual MacDonald^1^, Olga Kondrashova^2^, Khoa Tran^1,2,3^, Katia Nones^2^, Lambros T. Koufariotis^2^, Cameron Bean^1^, John V. Pearson^2^, Maciej Trzaskowski^1*^, Nicola Waddell^2*^

**Supplementary Figures**


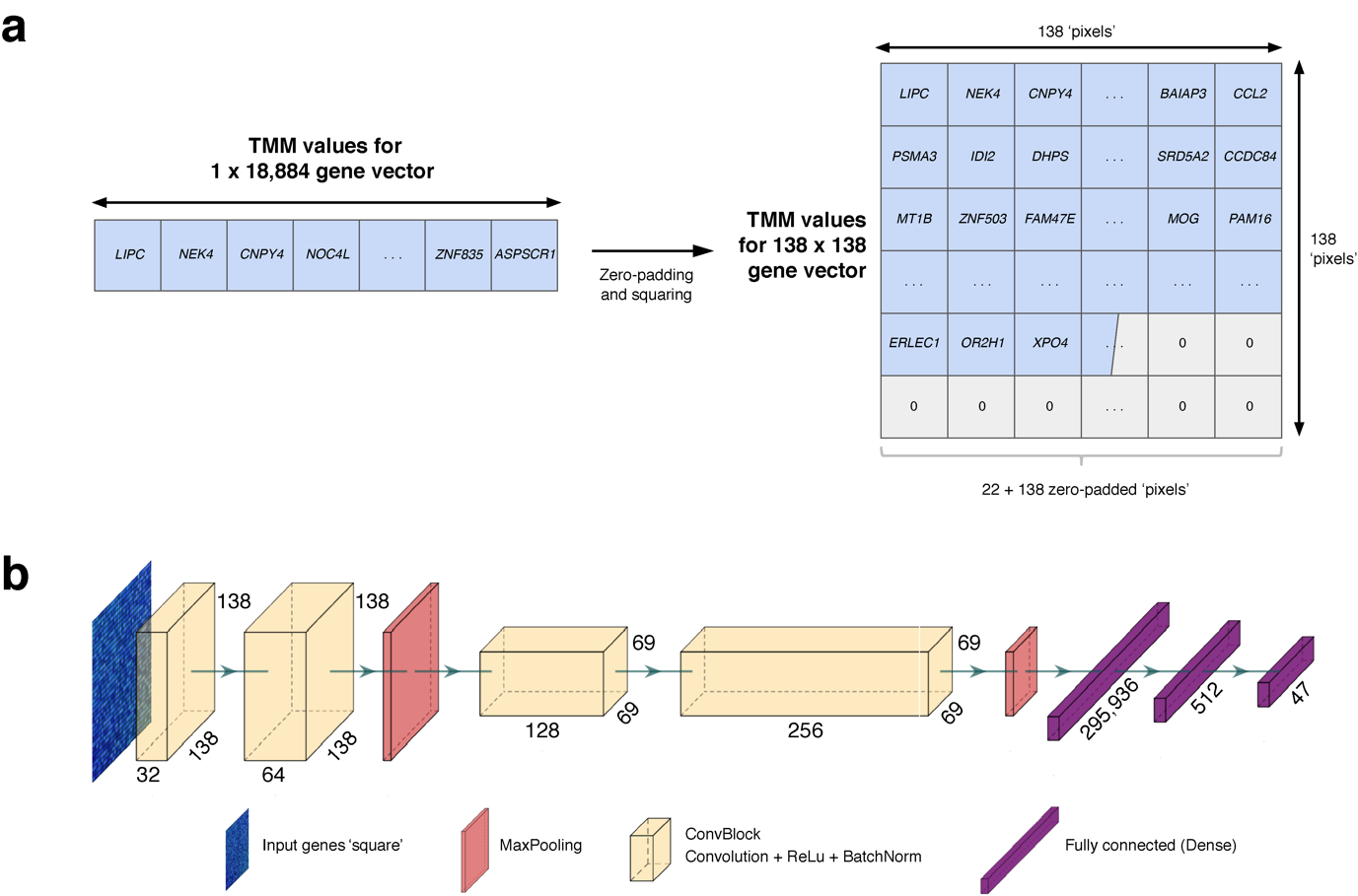


**Supplementary Fig 1. Technical workflow to calculate SHapley Additive exPlanations (SHAP) values and convolutional neural network (CNN) model architecture. a** Process of converting the gene-vector into a matrix suitable for input to the CNN. Note the need to pad the structure with zero values to complete the square. **b** CNN topology, where input data are gene expression values in the form of a square matrix for a given sample. The hidden layer network outputs one of 47 classes (tissue type). Each convolution block (ConvBlock) is a stack of layers comprising a convolution layer, activation layer (Rectified Linear Unit [ReLU]), and normalisation layer (Batch Normalization [BatchNorm]). MaxPooling refers to a layer which down-samples the input representation, while the fully connected (dense) layer flattens the preceding matrix into a single vector.


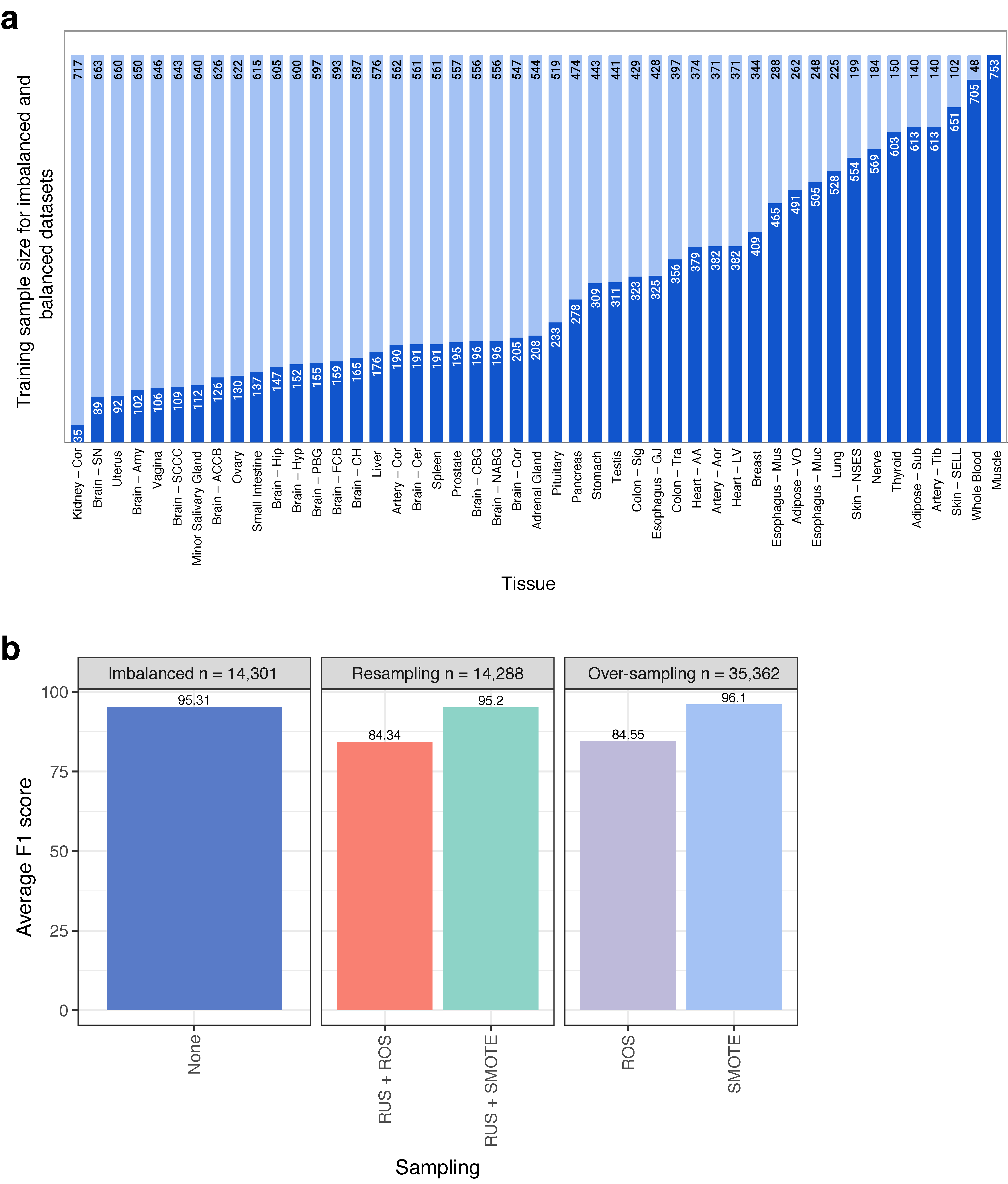


**Supplementary** **Fig 2.** **Comparing the performance of different balancing techniques.** **a** The number of training samples per class for the imbalanced (dark blue) and balanced (light blue) datasets. **b** Bar chart showing the recall values based on Trimmed Mean of M-values (TMM) normalised data and macro average F1 (harmonic mean of precision and recall) for the imbalanced and balanced datasets. ROS: Random Over-Sampling; RUS: Random Under-Sampling; SMOTE: Synthetic Minority Over-sampling Technique.


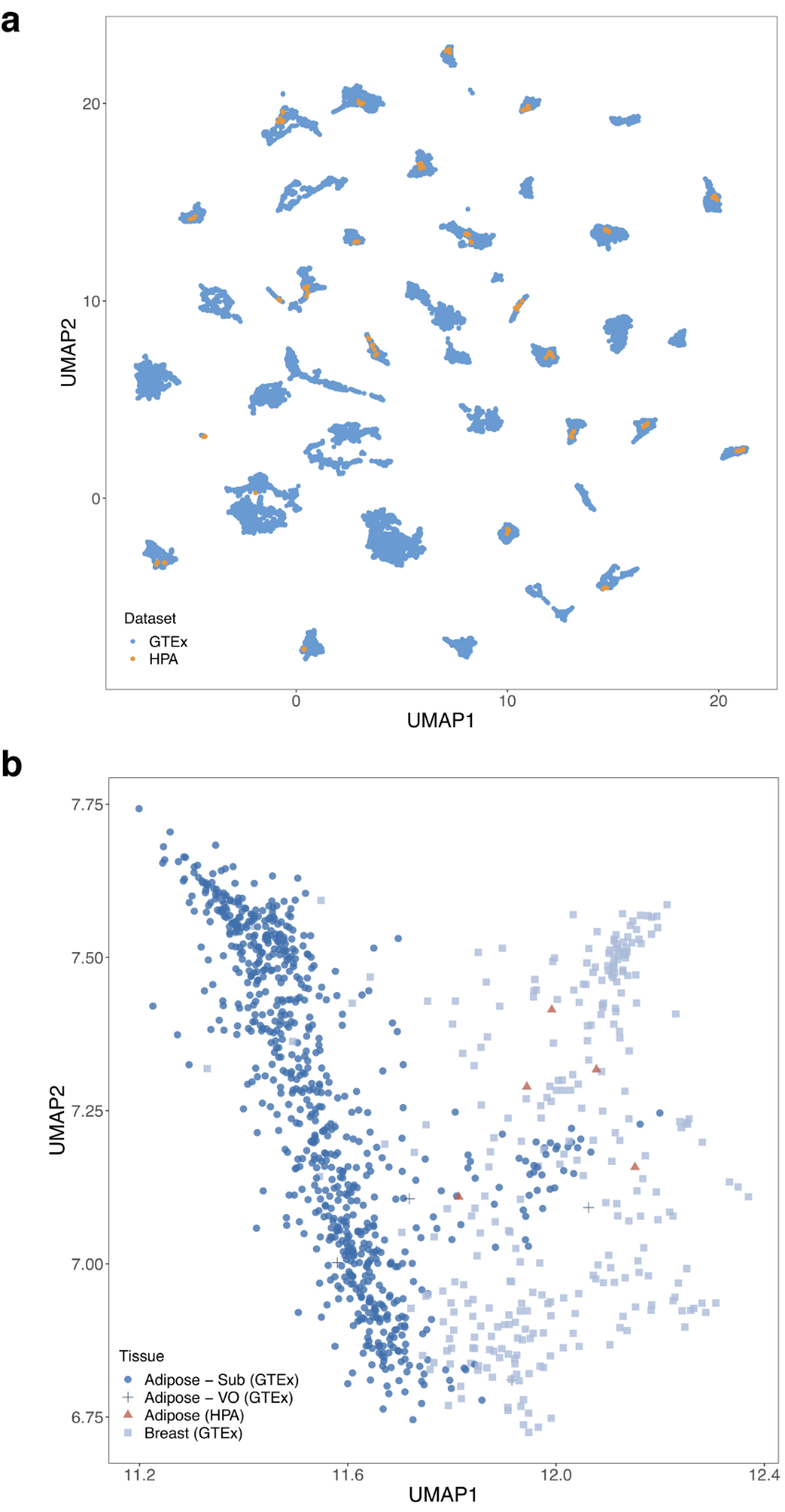


**Supplementary Fig 3. UMAP of balanced training GTEx samples and HPA independent dataset using all genes. a** UMAP of all SMOTE-balanced training GTEx samples (n = 35,362) and HPA samples (n = 126). **b** Zoomed in UMAP showing HPA adipose samples clustering with breast tissue samples rather than subcutaneous adipose tissue samples.


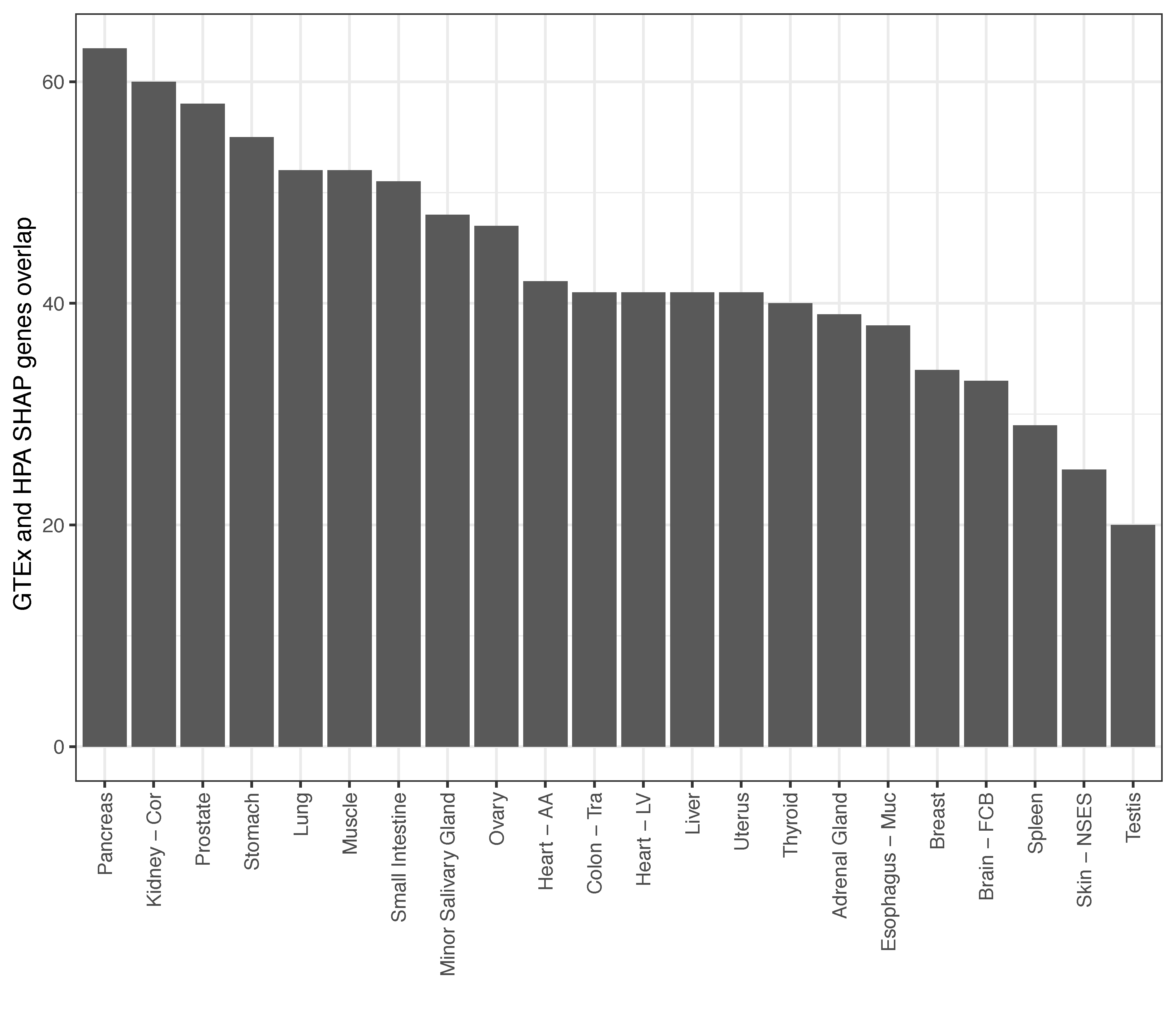


**Supplementary Fig 4. Overlap of SHAP genes for GTEx held-out test samples and HPA samples across classes present in the HPA independent dataset.**


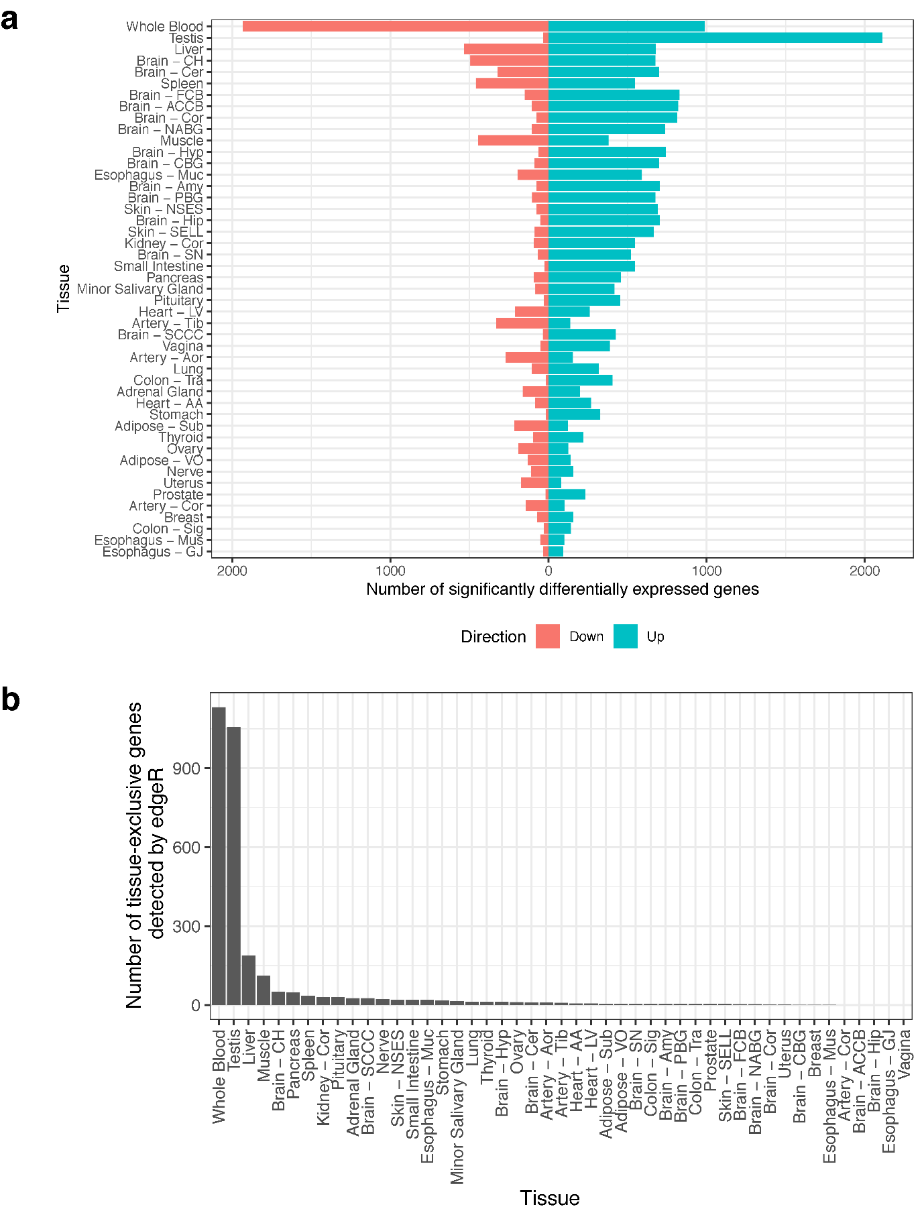


**Supplementary Fig 5. The number of significantly differentially expressed genes within each tissue type. a** Bar plots of the number of down- (red) and up-regulated (teal) genes when performing differential expression analysis between the samples of a given tissue against all samples from the remaining tissues using edgeR. Genes were considered significant if false discovery rate (FDR) > 0.01 and log2 fold change (logFC) was > 4. **b** The number of significantly differentially expressed tissue-exclusive genes per tissue type. Artery – Cor, Brain – ACCB, Brain – Hip, Esophagus – GJ, and Vagina had no tissue-exclusive gene expression, while Brain – CBG, Breast, and Esophagus – Mus had only one tissue-exclusive expressed gene.


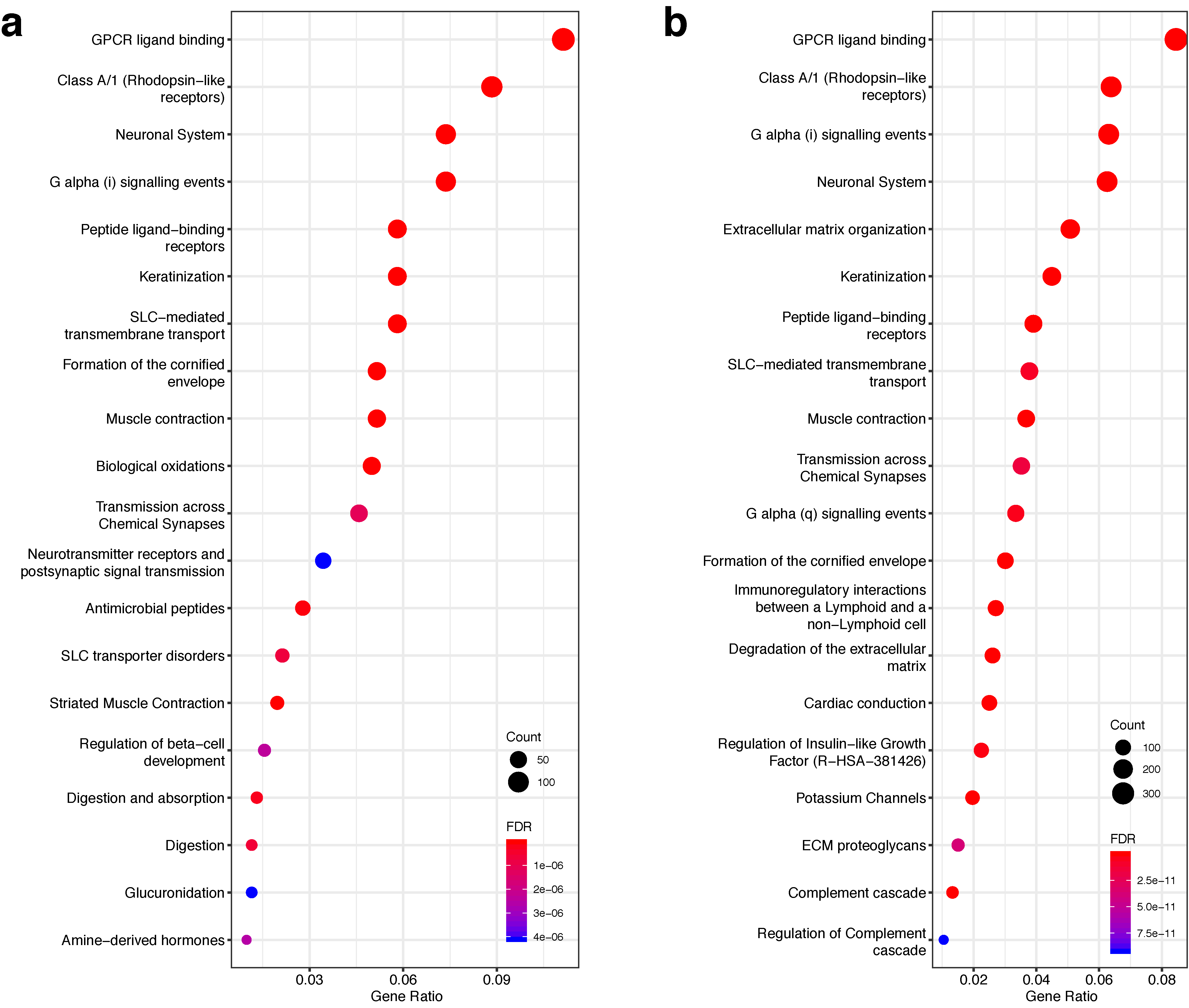


**Supplementary Fig 6. Reactome pathway analysis of genes identified from the CNN model using SHAP and from edgeR.** **a** Reactome pathway analysis of SHAP genes (2,423 genes). **b** Reactome pathway analysis of edgeR genes (7,854 genes).


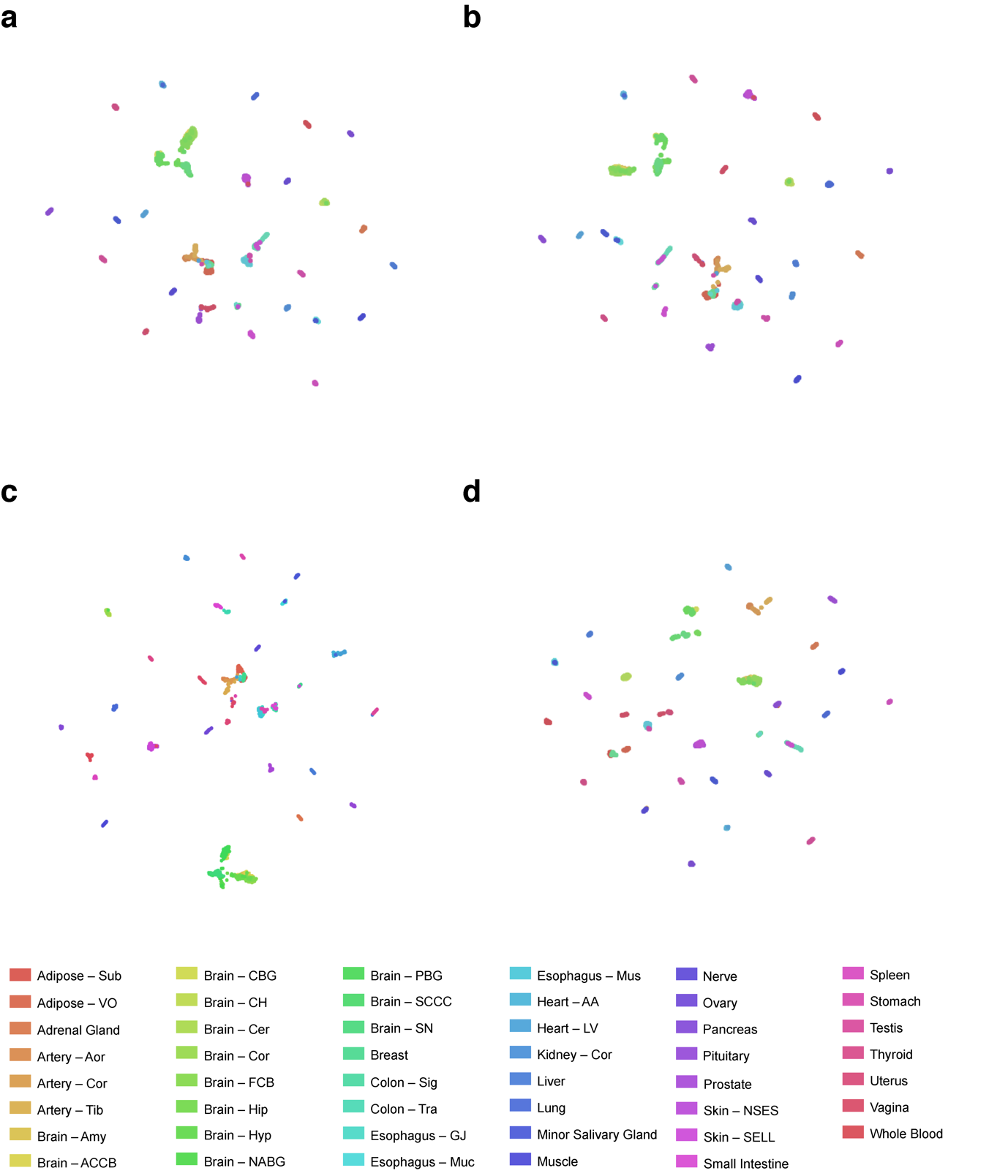


**Supplementary Fig 7. UMAPs of held-out GTEx samples using different gene subsets.** UMAP of held-out GTEx samples based on TMM normalised gene expression data for **a** all genes (18,884 genes), **b** edgeR genes (7,854 genes), **c** random genes (2,423 genes; representative UMAP from 10 separate runs), and **d** SHAP genes (2,423 genes). Tissue types represented by colours (bottom legend).


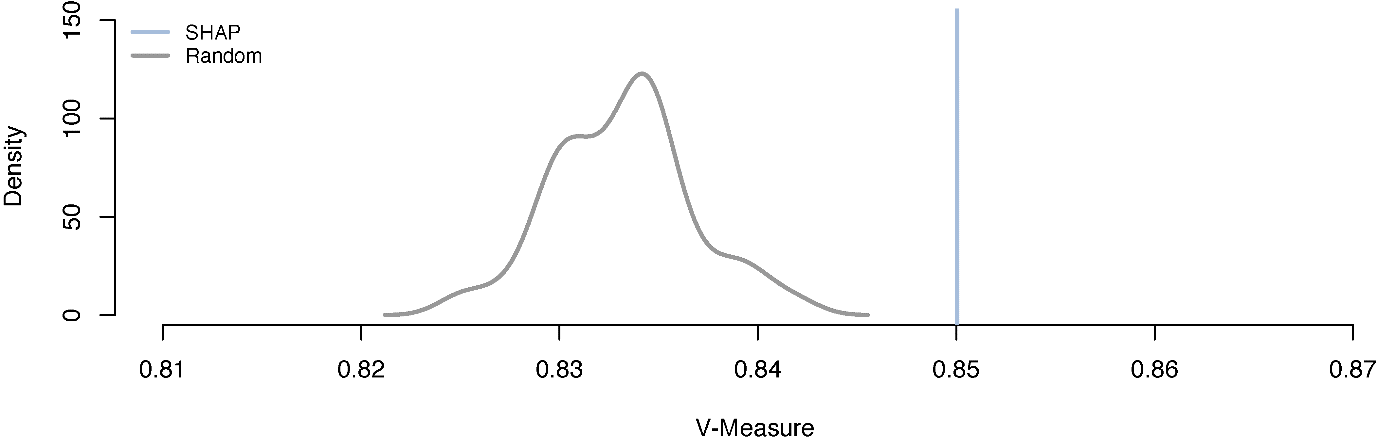


**Supplementary Fig 8. Comparison of tissue clustering performance using SHAP genes or random gene subset of equivalent size.** ‘Null’ distribution of V-Measure means was build using 100 random gene subsets of 2,423 genes. Mean V-Measure for SHAP genes is shown.

**Supplementary Tables**

**Supplementary Table 1. Distribution of tissue types in the GTEx dataset.**

| GTEx Tissue | Abbreviation | Total Sample Number | Train Sample Number | Test Sample Number |
| --- | --- | --- | --- | --- |
| Adipose – Subcutaneous | Adipose – Sub | 663 | 613 | 50 |
| Adipose – Visceral (Omentum) | Adipose – VO | 541 | 491 | 50 |
| Adrenal Gland | Adrenal Gland | 258 | 208 | 50 |
| Artery – Aorta | Artery – Aor | 432 | 382 | 50 |
| Artery – Coronary | Artery – Cor | 240 | 190 | 50 |
| Artery – Tibial | Artery – Tib | 663 | 613 | 50 |
| Brain – Amygdala | Brain – Amy | 152 | 102 | 50 |
| Brain – Anterior cingulate cortex (BA24) | Brain – ACCB | 176 | 126 | 50 |
| Brain – Caudate (basal ganglia) | Brain – CBG | 246 | 196 | 50 |
| Brain – Cerebellar Hemisphere | Brain – CH | 215 | 165 | 50 |
| Brain – Cerebellum | Brain – Cer | 241 | 191 | 50 |
| Brain – Cortex | Brain – Cor | 255 | 205 | 50 |
| Brain – Frontal Cortex (BA9) | Brain – FCB | 209 | 159 | 50 |
| Brain – Hippocampus | Brain – Hip | 197 | 147 | 50 |
| Brain – Hypothalamus | Brain – Hyp | 202 | 152 | 50 |
| Brain – Nucleus accumbens (basal ganglia) | Brain – NABG | 246 | 196 | 50 |
| Brain – Putamen (basal ganglia) | Brain – PBG | 205 | 155 | 50 |
| Brain – Spinal cord (cervical c-1) | Brain – SCCC | 159 | 109 | 50 |
| Brain – Substantia nigra | Brain – SN | 139 | 89 | 50 |
| Breast – Mammary Tissue | Breast | 459 | 409 | 50 |
| Colon – Sigmoid | Colon – Sig | 373 | 323 | 50 |
| Colon – Transverse | Colon – Tra | 406 | 356 | 50 |
| Esophagus – Gastroesophageal Junction | Esophagus – GJ | 375 | 325 | 50 |
| Esophagus – Mucosa | Esophagus – Muc | 555 | 505 | 50 |
| Esophagus – Muscularis | Esophagus – Mus | 515 | 465 | 50 |
| Heart – Atrial Appendage | Heart – AA | 429 | 379 | 50 |
| Heart – Left Ventricle | Heart – LV | 432 | 382 | 50 |
| Kidney – Cortex | Kidney – Cor | 85 | 35 | 50 |
| Liver | Liver | 226 | 176 | 50 |
| Lung | Lung | 578 | 528 | 50 |
| Minor Salivary Gland | Minor Salivary Gland | 162 | 112 | 50 |
| Muscle – Skeletal | Muscle | 803 | 753 | 50 |
| Nerve – Tibial | Nerve | 619 | 569 | 50 |
| Ovary | Ovary | 180 | 130 | 50 |
| Pancreas | Pancreas | 328 | 278 | 50 |
| Pituitary | Pituitary | 283 | 233 | 50 |
| Prostate | Prostate | 245 | 195 | 50 |
| Skin – Not Sun Exposed (Suprapubic) | Skin – NSES | 604 | 554 | 50 |
| Skin – Sun Exposed (Lower leg) | Skin – SELL | 701 | 651 | 50 |
| Small Intestine – Terminal Ileum | Small Intestine | 187 | 137 | 50 |
| Spleen | Spleen | 241 | 191 | 50 |
| Stomach | Stomach | 359 | 309 | 50 |
| Testis | Testis | 361 | 311 | 50 |
| Thyroid | Thyroid | 653 | 603 | 50 |
| Uterus | Uterus | 142 | 92 | 50 |
| Vagina | Vagina | 156 | 106 | 50 |
| Whole Blood | Whole Blood | 755 | 705 | 50 |
|  |  | **16651** | **14301** | **2350** |

**Supplementary Data Description**

**Supplementary Data 1. CNN class predictions for the independent HPA dataset.**

**Supplementary Data 2. The top 103 ranks of genes ranked by median SHAP value for each tissue type – GTEx held-out test samples: a** The top 103 ranks per tissue in long format, where each column represents a unique variable: tissue type, rank, gene name, and median SHAP value. **b** The top 103 ranks in wide format, where tissue type is spread across the columns, ranks are in rows, and gene names are coloured to visualise unique genes in expanding top n ranks. Dark blue denotes a gene's first appearance in the order of ranks; light blue denotes a gene's first appearance if represented in more than one tissue at the same rank; and no fill denotes all subsequent appearances of a gene.

**Supplementary Data 3. The top 103 ranks of genes ranked by median SHAP value for each tissue type – HPA independent dataset: a** The top 103 ranks per tissue in long format, where each column represents a unique variable: tissue type, rank, gene name, and median SHAP value. **b** he top 103 ranks in wide format, where tissue type is spread across the columns, ranks are in rows, and gene names are coloured to visualise unique genes in expanding top n ranks. Dark blue denotes a gene's first appearance in the order of ranks; light blue denotes a gene's first appearance if represented in more than one tissue at the same rank; and no fill denotes all subsequent appearances of a gene.

**Supplementary Data 4. Protein-protein interaction network analysis using STRING for SHAP genes and tissue-exclusive genes.** * denotes significant p-value (<0.05).

**Supplementary Data 5. Enriched GO biological processes, KEGG and Reactome pathways for SHAP genes across all tissues. a** Summary and **b** full list.

**Supplementary Data 6. Enriched GO biological processes, KEGG and Reactome pathways for tissue-exclusive genes across all tissues. a** Summary and **b** full list.
